# Supplementary material for: Variability of the Indian Ocean Dipole post-2100 reverses to a reduction despite persistent global warming
Source: Nat Commun. 2024 Jun 12;15:5023. doi: 10.1038/s41467-024-49401-y (PMC11169280; doi:10.1038/s41467-024-49401-y)
Supplement: Supplementary file 1 — Supplementary Information [file 41467_2024_49401_MOESM1_ESM.pdf]

Supplementary Information for

Decreased variability of the Indian Ocean Dipole after 2100 despite persistent global warming

Guojian Wang[1], Wenju Cai[*1,2,3,4,5], Agus Santoso[*1,6,7]

*Corresponding author: Wenju.Cai@csiro.au & a.santoso@unsw.edu.au

This PDF file includes:

Supplementary Figs. 1 to 12

Supplementary Table 1

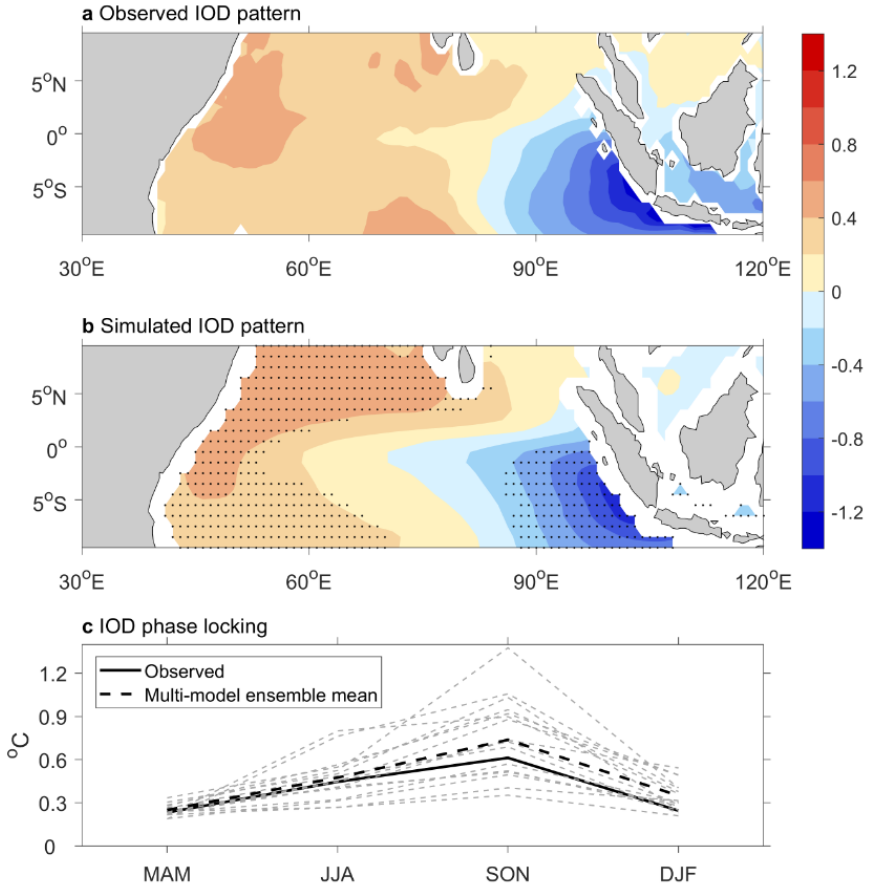

Supplementary Fig. 1 | Observed and simulated IOD pattern and phase locking over 1958-2022. a, The observed IOD pattern which is calculated as the SST regression pattern associated with SON DMI using ORA-s5. b, The same as a, but for multi-model ensemble mean of simulated IOD pattern. Dotted area indicates where at least 90% models agree on the sign of multi-model ensemble mean. c, Observed and simulated IOD phase locking as indicated by solid and dashed black curves, respectively. Dashed grey curves indicate individual models. Although the ensemble mean of SON amplitude is greater than observed, there is a lack of inter-model agreement on such bias with around half models simulating smaller amplitude than observation. Models used in present study can simulate reasonable IOD pattern, phase locking and magnitude.

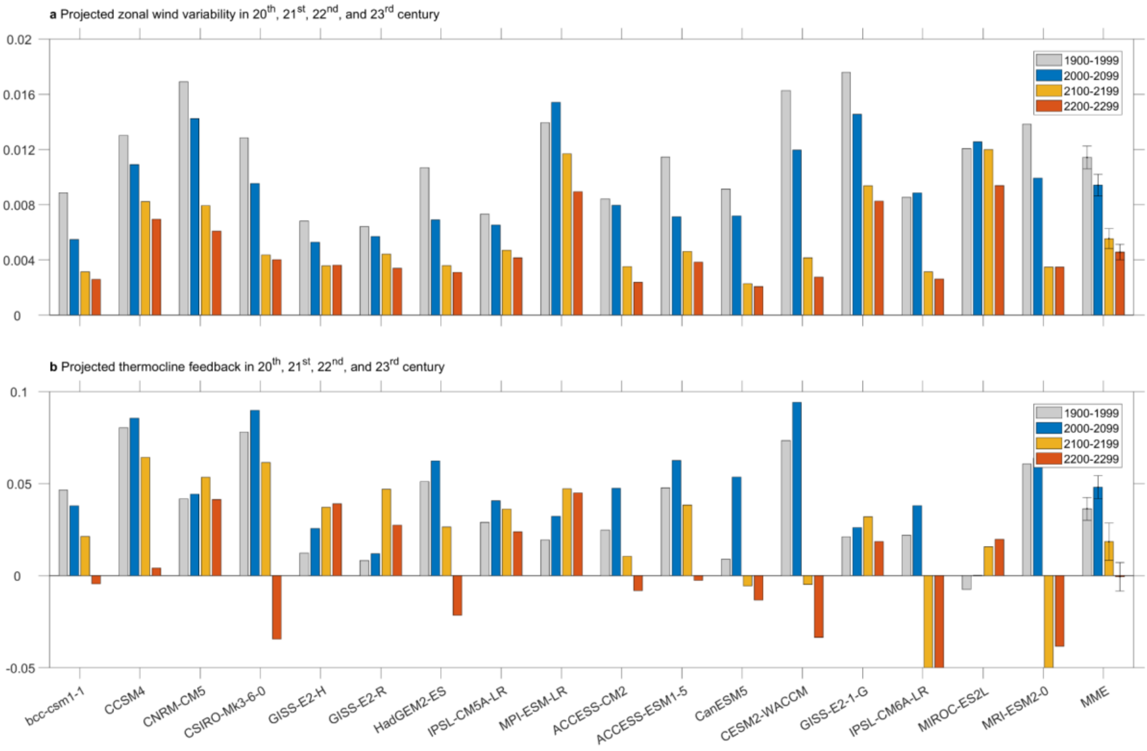

Supplementary Fig. 2 | Inter-model consensus on projected changes of zonal wind variability and the thermocline feedback from the 20th to the 23rd century. a, and b, The projected changes in zonal wind variability and the thermocline feedback, respectively. The error bar for the multi-model ensemble mean is a one-standard-deviation range based on a bootstrap test. Zonal wind variability deceases from the 20st century onward, but the thermocline feedback initially intensifies in the 21st century before weakens or even ceases to operate thereafter.

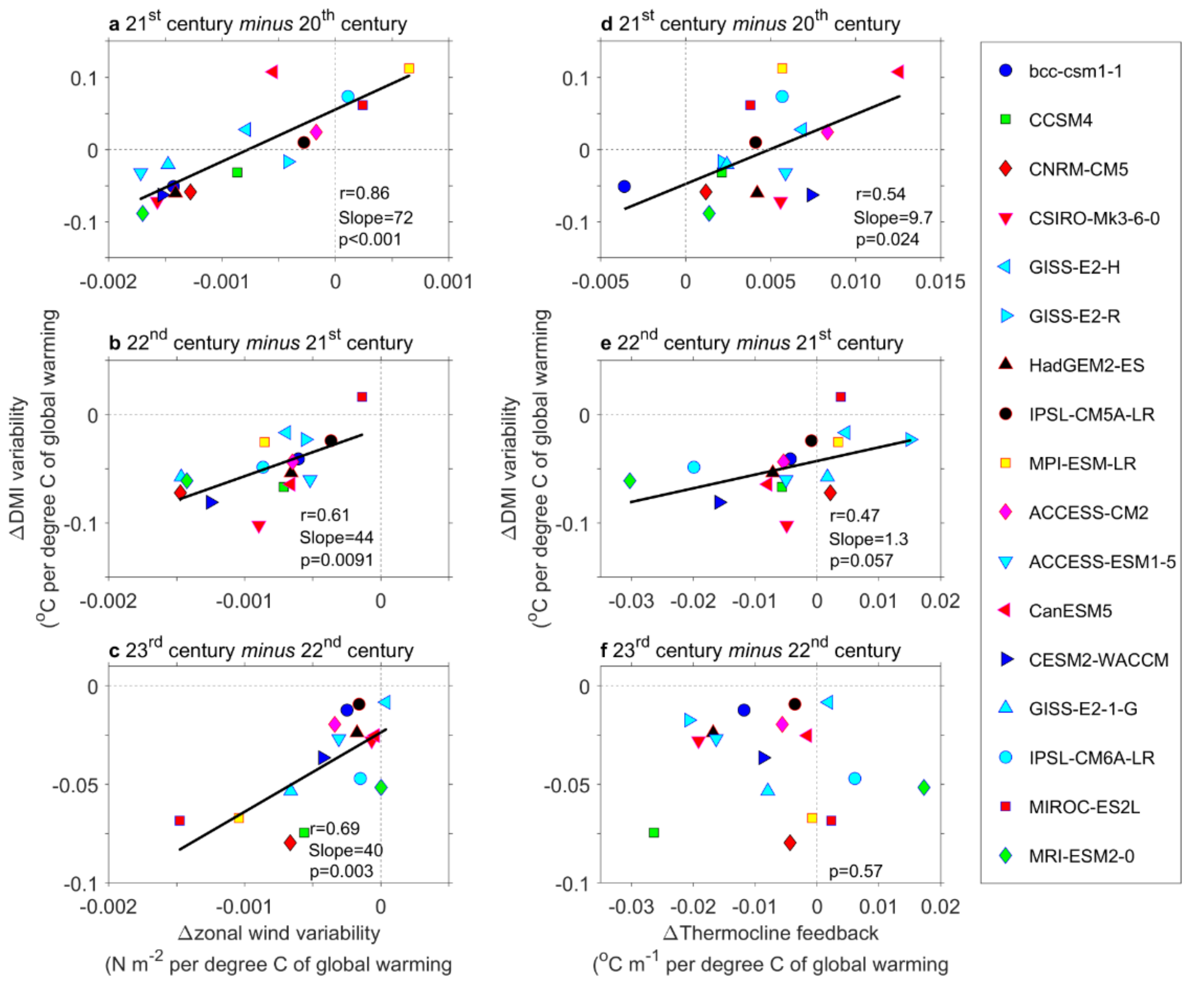

Supplementary Fig. 3 | Inter-model relationship in the projected changes between zonal wind variability and the thermocline feedback, and DMI SST variability. a, Projected changes in zonal wind variability against that in DMI variability from the 20th to the 21st century. The correlation coefficient, slope, and p-value are indicated. b and c, The same as a, but for projected changes from the 21st to the 22nd century, and from the 22nd to the 23rd century, respectively. d-f, The same as a-c, but for the inter-model relationship in projected changes between the thermocline feedback and DMI variability. The changes are scaled by the associated projected changes in the global mean temperature. Reduced zonal wind variability contributes to decreased DMI variability from the 20th century onward, but the intensified thermocline feedback in the 21st century contributes to an increase. However, after 2100 the thermocline feedback weakens or even ceases to operate, no longer influences DMI variability.

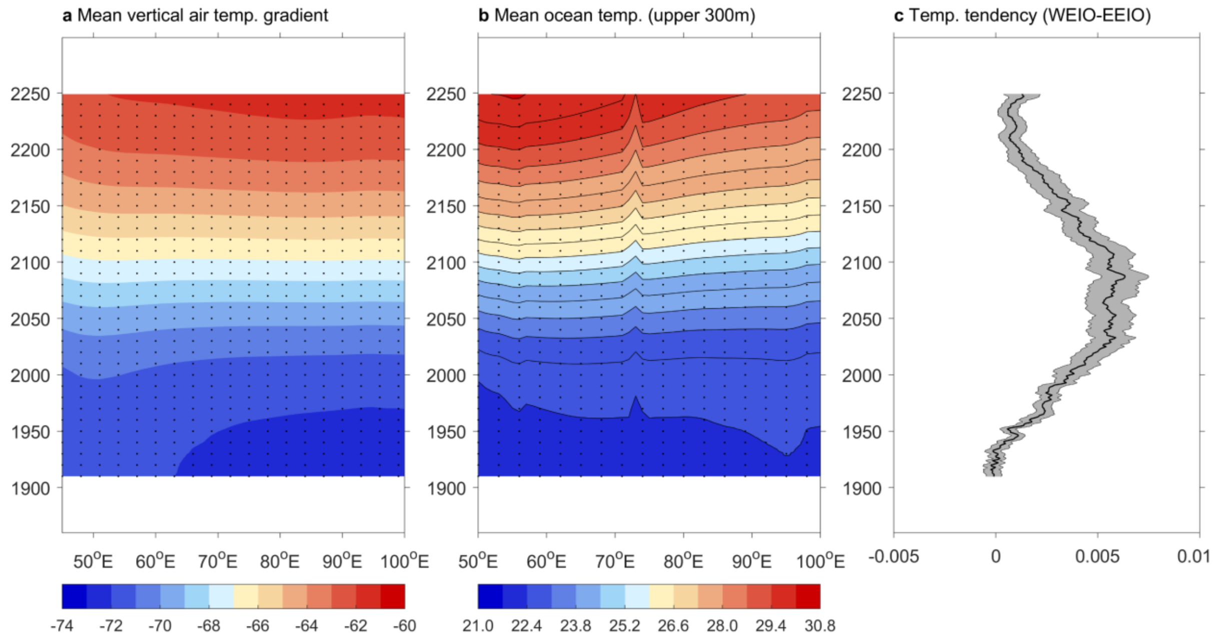

Supplementary Fig. 4 | The time evolution in the mean state of vertical atmospheric stability and subsurface oceanic warming in a 100-year running average. a, The multi-model ensemble mean of annual mean vertical air temperature gradient between 200hpa and 850hpa (200 hpa *minus* 850 hpa) in a 100-year running average. This is zonally averaged between 5ºS and 5ºN. The dotted area indicates regions where the mean vertical air temperature gradient is statistically different from the mean over the 1860-1900 above the 90% confidence level based on a student's *t*-test. b, The multi-model ensemble mean of annual mean ocean temperature averaged between 5ºS and 5ºN and over upper 300m depth. The dotted area indicates regions where the mean climatological temperature is statistically different from the mean over the 1860-1900 above the 90% confidence level based on a student's t-test. c, The difference in the ocean temperature warming tendency between the WEIO and the EEIO. The black curve indicates the multi-model ensemble mean and the grey shadows indicate the one-standard-deviation range based on a Bootstrap test. For mean state change in the atmosphere, the vertical temperature difference is persistently decreasing at all longitudes, i.e., the atmosphere is more stable; for mean state change in the ocean, oceanic warming extends towards the east after 2100, instead of towards the west as before 2100, leading to the increase in the eastern warming rate, hampering the shoaling of the EEIO thermocline.

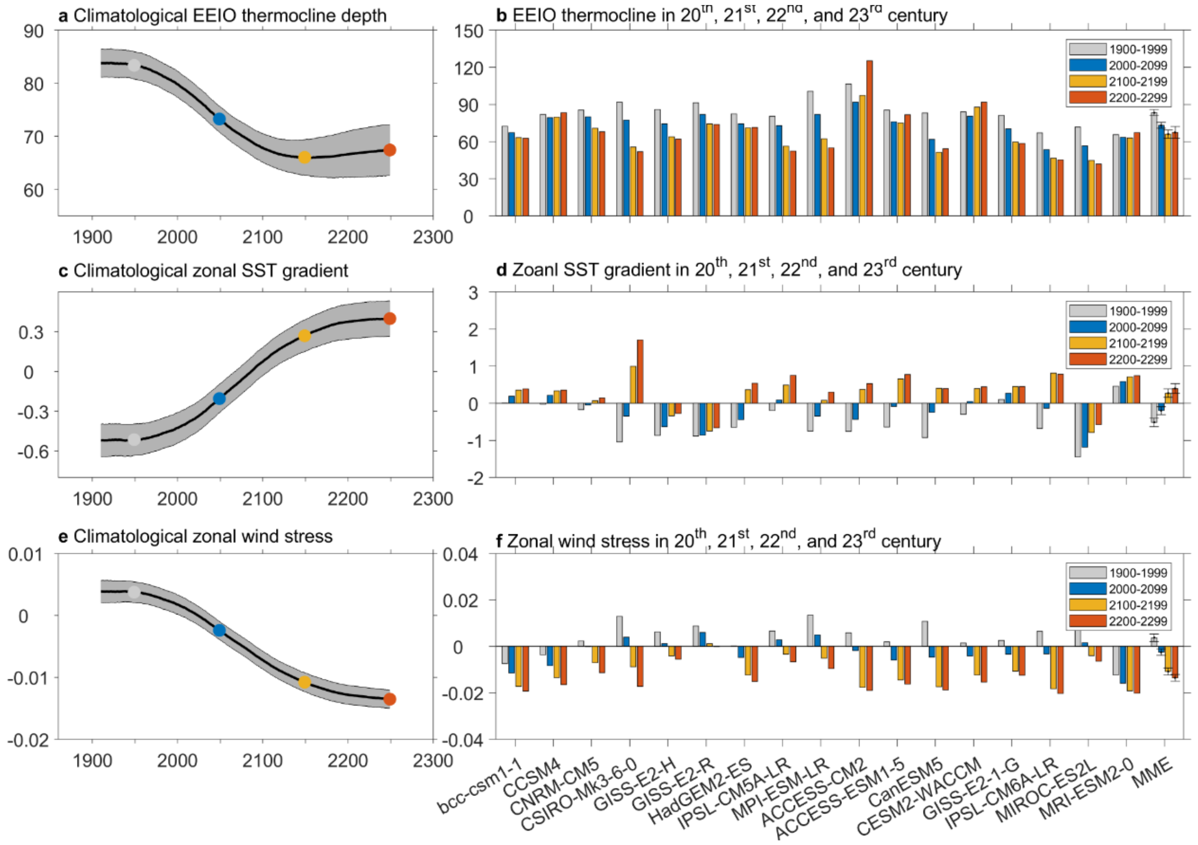

Supplementary Fig. 5 | Time evolution of annual mean thermocline depth, zonal SST gradient, and zonal wind stress. a, c, and e, Annual mean of the EEIO thermocline, zonal SST gradient between the WEIO and the EEIO, and CTIO zonal wind stress, respectively. The black curve indicates the multi-model ensemble mean and the grey shadows indicate the one-standard-deviation range based on a bootstrap test (see ‘Statistical significance test’ in Methods). b, d, and f, Annual mean of the EEIO thermocline, zonal SST gradient, and CTIO zonal wind stress, respectively, over the 20th, the 21st, the 22nd, and the 23rd centuries for each model corresponding to values indicated by the grey, blue, yellow, and orange dots in a. The error bar with the multi-model ensemble mean is one-standard-deviation-range based on bootstrap. From the 22nd to the 23rd century the EEIO thermocline shows little further shallowing, with correspondingly little change in the zonal SST gradient, or easterlies.

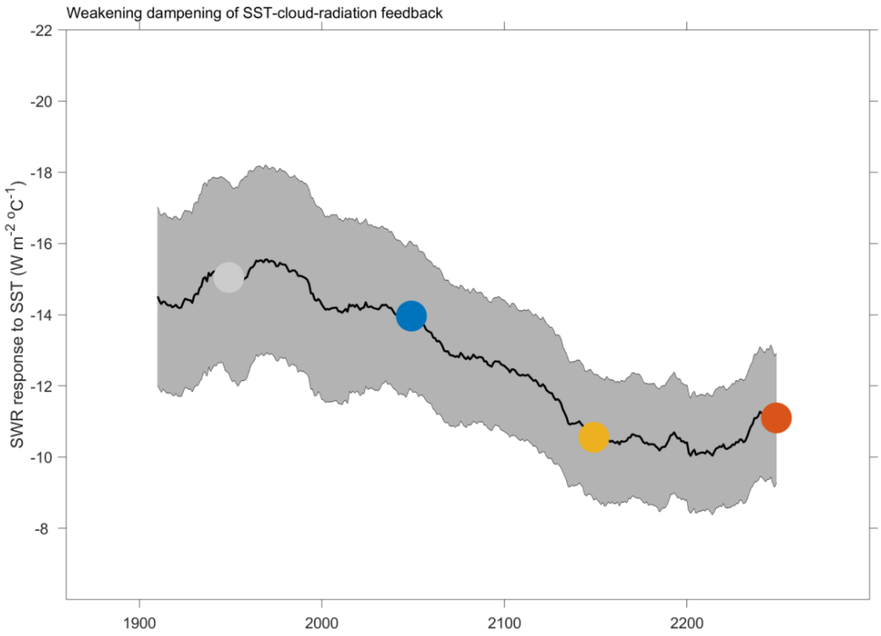

Supplementary Fig. 6 | Time evolution in surface shortwave radiation (SWR) response to SST anomalies towards 2300 over the eastern equatorial Indian Ocean. It is calculated as the regression coefficient of SWR onto SST anomalies for each of the 100-year window focusing on SON season. The SWR is positive downward. The black curve indicates the multi-model ensemble mean and the grey shadows indicate the one-standard-deviation range based on a Bootstrap test. The grey, blue, yellow, and orange dots indicate values over the 20th, the 21st, the 22nd, and the 23rd centuries. The weakening in the IOD thermal damping would lead to increased IOD SST variability.

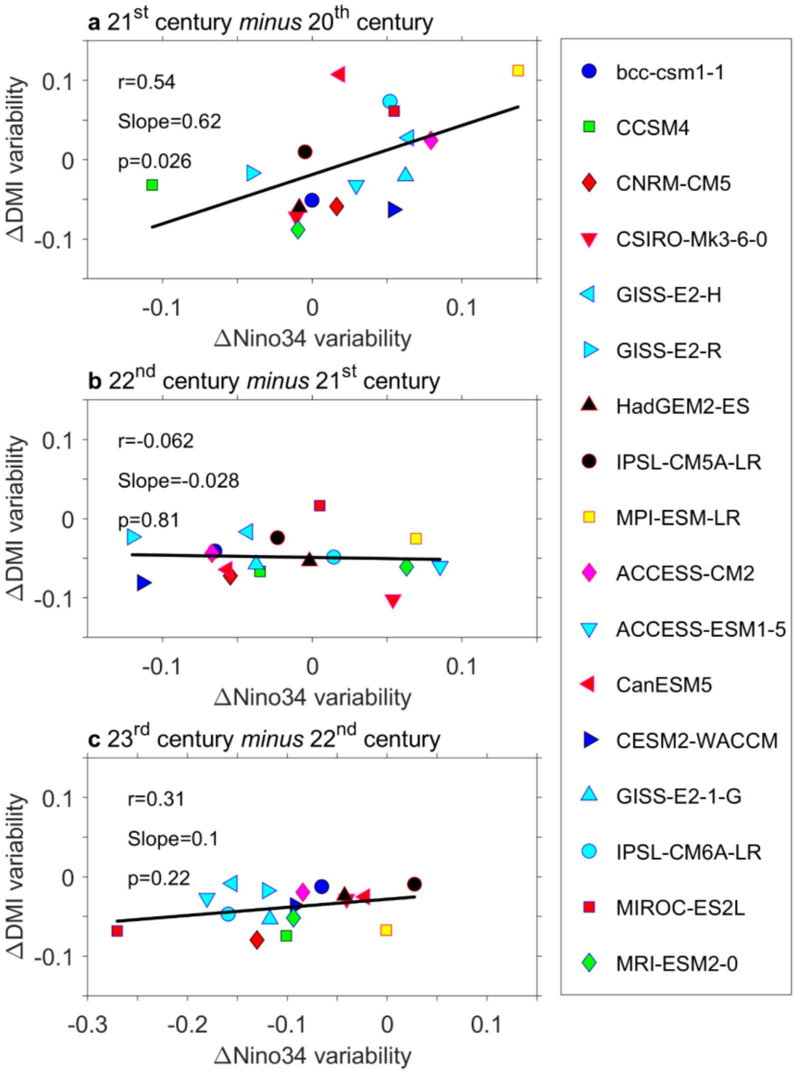

Supplementary Fig. 7 | Inter-model relationship in the projected changes between Niño3.4 SST variability and DMI SST variability. a, Projected changes in Niño3.4 (5° S–5° N, 170° W–120° W) SST variability against that in DMI variability from the 20th to the 21st century focusing on SON season. The correlation coefficient, slope, and p-value are indicated. b and c, The same as a, but for projected changes from the 21st to the 22nd century, and from the 22nd to the 23rd century, respectively. The changes are scaled by the associated projected changes in the global mean temperature. Changes in ENSO variability after the 21st century does not contribute to changes in the DMI.

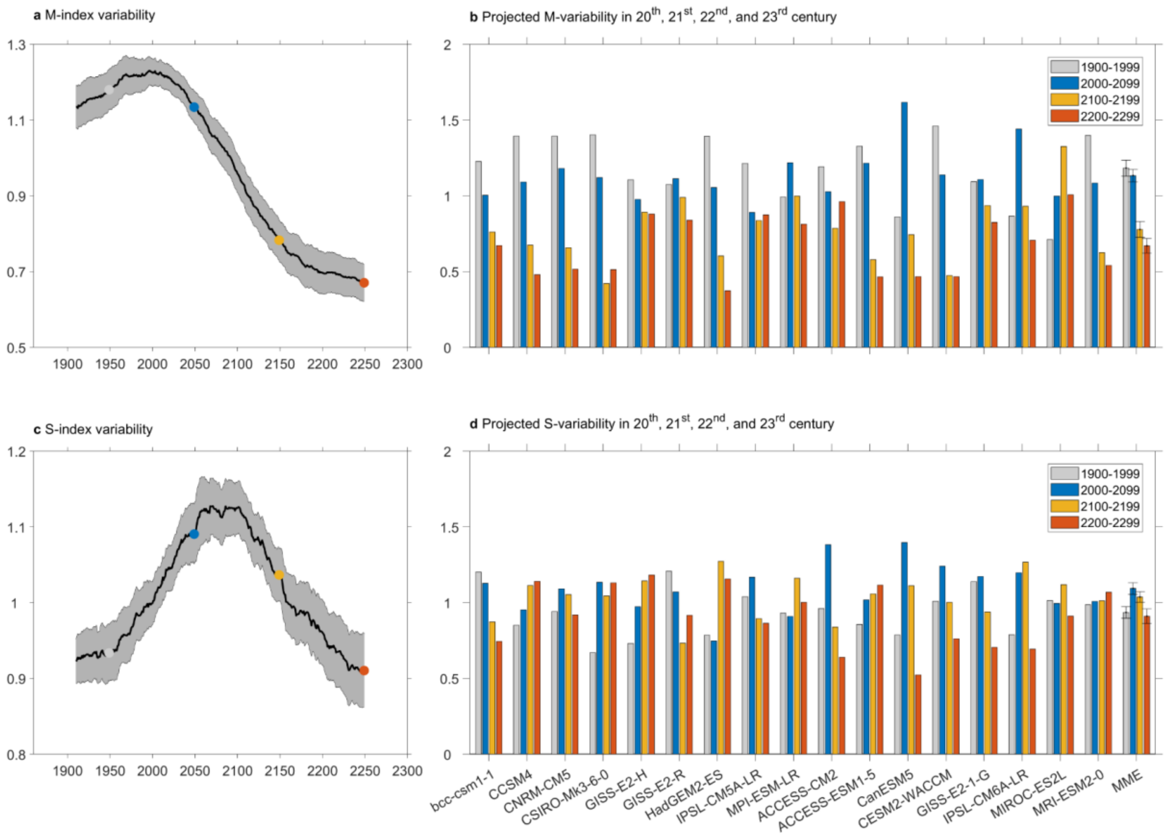

Supplementary Fig. 8 | Time evolution of strong-pIOD SST variability and moderate-pIOD SST variability. a and c, September, October, and November (SON) SST variability for M-index and S-index, respectively. The black curve indicates the multi-model ensemble mean and the grey shadows indicate the one-standard-deviation range based on a bootstrap test (see 'Statistical significance test' in Methods). b, M-index variability over the 20th, the 21st, the 22nd, and the 23rd centuries for each model corresponding to values indicated by the grey, blue, yellow, and orange dots in a. The error bar with the multi-model ensemble mean is one-standard-deviation-range based on bootstrap. d, The same as b, but for S-index variability. Before the 21st century, moderate-pIOD decreases but strong-pIOD increases; thereafter both decrease.

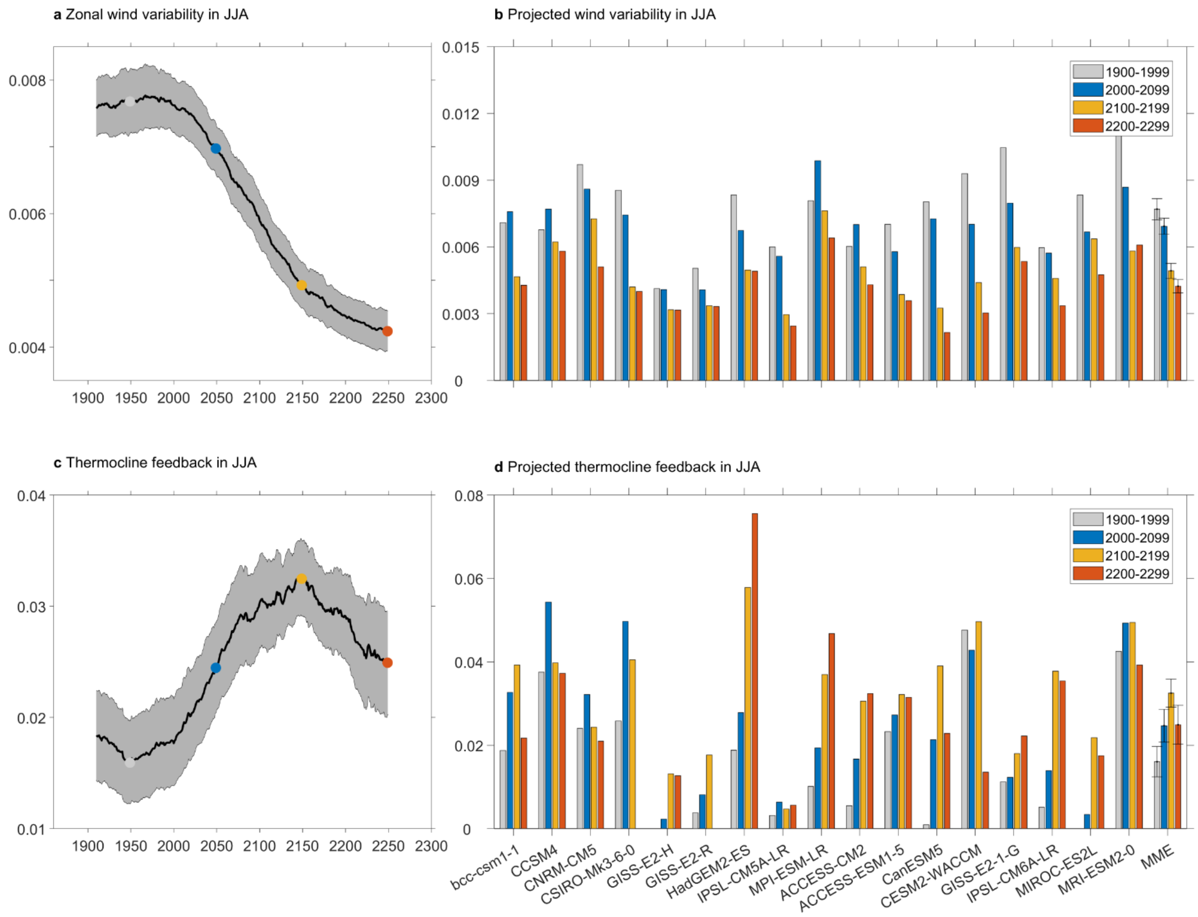

Supplementary Fig. 9 | Time evolution of zonal wind variability and the thermocline feedback in JJA. a and b, c and d, The same as Fig. 1a and Fig. 1b, but for zonal wind variability and the thermocline feedback in JJA, respectively. Reduced zonal wind variability contributes to decreased JJA DMI variability from the 20th century onward, but the intensified thermocline feedback in the 21st century contributes to an increase. However, after 2200 the thermocline feedback weakens.

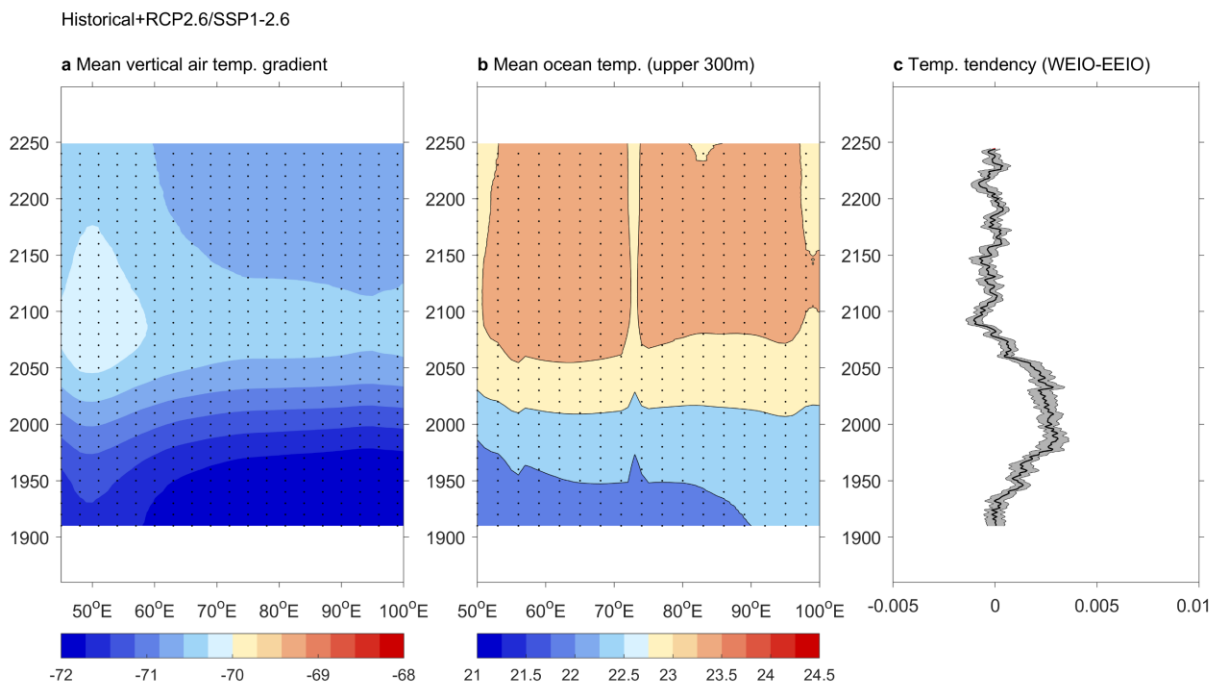

Supplementary Fig. 10 | The time evolution in the mean state of vertical atmospheric stability and subsurface oceanic warming in a 100-year running average forced under the low CO2 emission scenario. a, The multi-model ensemble mean of annual mean vertical air temperature gradient between 200hpa and 850hpa (200 hpa minus 850 hpa) in a 100-year running average. This is zonally averaged between 5ºS and 5ºN. The dotted area indicates regions where the mean vertical air temperature gradient is statistically different from the mean over the 1860-1900 above the 90% confidence level based on a student's t-test. b, The multi-model ensemble mean of annual mean ocean temperature averaged between 5ºS and 5ºN and over upper 300m depth. The dotted area indicates regions where the mean climatological temperature is statistically different from the mean over the 1860-1900 above the 90% confidence level based on a student's t-test. c, The difference in the ocean temperature warming tendency between the WEIO and the EEIO. The black curve indicates the multi-model ensemble mean and the grey shadows indicate the one-standard-deviation range based on a Bootstrap test. The increasing atmospheric stability and oceanic warming both stabilise under the low CO2 emission scenario after the 21st century.

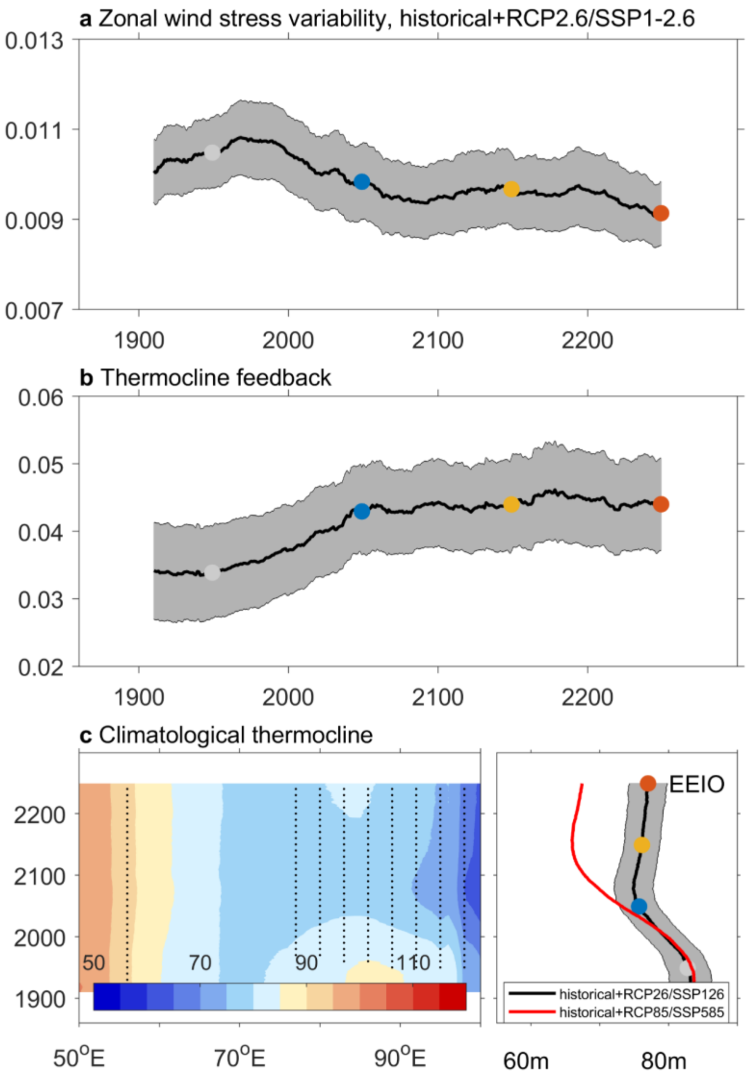

Supplementary Fig. 11 | Mechanism of IOD time evolution under historical and RCP2.6/SSP1-2.6 emission scenarios. a, b, and c, The same as Fig. 2a, Fig. 2b, and Fig. 2c, respectively, but using data forced under historical and RCP2.6/SSP1-2.6 emission scenario. In the right panel of c, the time evolution of EEIO mean thermocline depth is plotted on the right side with black and red curves indicating the multi-model ensemble mean under low, and high CO2 emission scenario, respectively. In contrast to the RCP8.5/SSP5-8.5, both zonal wind variability and thermocline feedback stabilise after the 21st century.

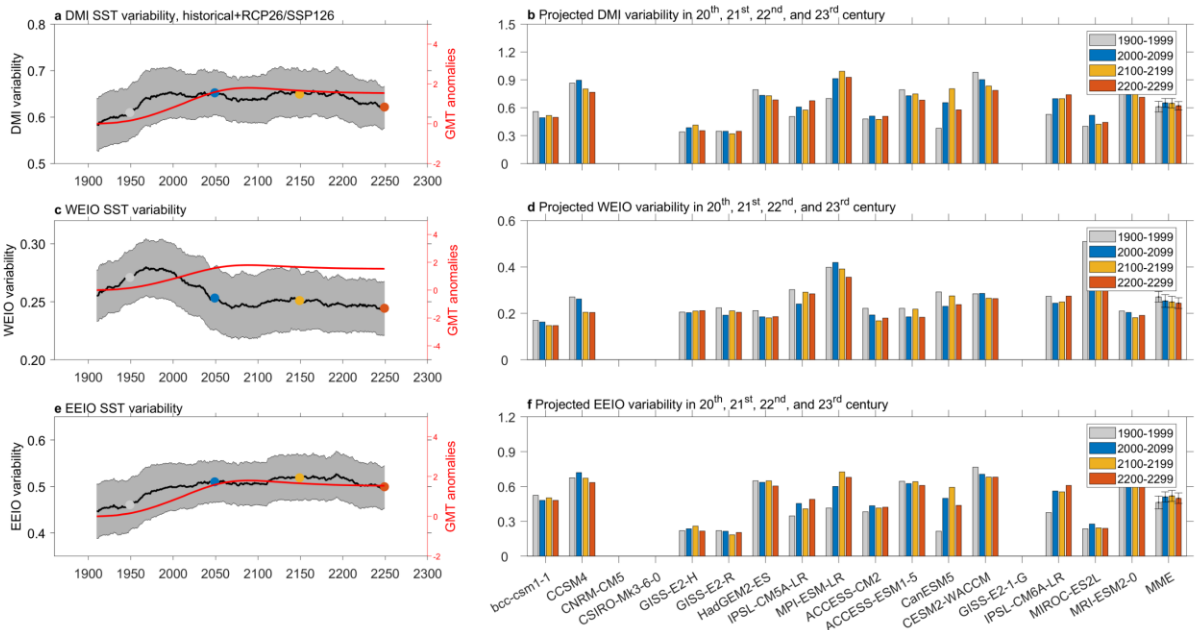

Supplementary Fig. 12 | Time evolution of IOD variability in a 100-year sliding window from 1860 to 2300 forced under historical and RCP2.6/SSP1-2.6 emission scenarios. a, September, October, and November (SON) SST variability of the Dipole Mode Index (DMI), defined as the difference in Sea Surface Temperature (SST) anomalies between the Western Equatorial Indian Ocean (WEIO; 50ºE-70ºE, 10ºS-10ºN) and the Eastern Equatorial Indian Ocean (EEIO; 90ºE-110ºE, 10ºS-0º). The black curve indicates the multi-model ensemble mean and the grey shadows indicate the one-standard-deviation range based on a bootstrap test (see 'Statistical significance test' in Methods). The red curve shows the multi-model ensemble mean of global mean temperature (GMT) in a 100-year running average, and then subtracting the mean GMT of the first 100-year window. b, DMI variability over the 20th, the 21st, the 22nd, and the 23rd centuries for each model corresponding to values indicated by the grey, blue, yellow, and orange dots in a. The error bar with the multi-model ensemble mean is one-standard-deviation-range based on bootstrap. c and d, e and f, The same as a and b, but for WEIO SST variability and EEIO SST variability, respectively. In contrast to the RCP8.5/SSP5-8.5, SST variability across the tropical Indian Ocean stabilises after the 21st century.

Supplementary Table 1 | Output availability in the 17 CMIP5 and CMIP6 models that are utilized in the present study. Those 17 models are selected due to the availability of long-term future projection towards the end of the 23rd century under the business-as-usual emission scenario (2006-2300 for CMIP5 RCP8.5 and 2015-2300 for CMIP6 SSP5-8.5). Monthly data of ocean temperature (thetao), sea surface temperature (sst), surface temperature (ts), atmosphere temperature (ta), zonal wind stress (tauu), and surface shortwave radiation (swr) are used. We also used data but forced under the low CO2 emission scenario, i.e., CMIP5 RCP2.6 and CMIP6 SSP1-2.6.

	Models	Realization	Data available for RCP8.5/SSP5-8.5	Data available for RCP2.6/SSP1-2.6
1	bcc-csm1-1	r1i1p1	thetao, sst, ts, ta, tauu, swr	thetao, sst, ts, ta, tauu, swr
2	CCSM4	r1i1p1	thetao, sst, ts, ta, tauu, swr	thetao, sst, ts, ta, tauu, swr
3	CNRM-CM5	r1i1p1	thetao, sst, ts, ta, tauu, swr	
4	CSIRO-Mk3-6-0	r1i1p1	thetao, sst, ts, ta, tauu, swr	
5	GISS-E2-H	r1i1p1	thetao, sst, ts, ta, tauu, swr	thetao, sst, ts, ta, tauu, swr
6	GISS-E2-R	r1i1p1	thetao, sst, ts, ta, tauu, swr	thetao, sst, ts, ta, tauu, swr
7	HadGEM2-ES	r1i1p1	thetao, sst, ts, ta, tauu, swr	thetao, sst, ts, ta, tauu, swr
8	IPSL-CM5A-LR	r1i1p1	thetao, sst, ts, ta, tauu, swr	thetao, sst, ts, ta, tauu, swr
9	MPI-ESM-LR	r1i1p1	thetao, sst, ts, ta, tauu, swr	thetao, sst, ts, ta, tauu, swr
10	ACCESS-CM2	r1i1p1f1	thetao, sst, ts, ta, tauu, swr	thetao, sst, ts, ta, tauu, swr
11	ACCESS-ESM1-5	r1i1p1f1	thetao, sst, ts, ta, tauu, swr	thetao, sst, ts, ta, tauu, swr
12	CanESM5	r1i1p1f1	thetao, sst, ts, ta, tauu, swr	thetao, sst, ts, ta, tauu, swr
13	CESM2-WACCM	r1i1p1f1	thetao, sst, ts, ta, tauu, swr	thetao, sst, ts, ta, tauu, swr
14	GISS-E2-1-G	r1i1p1f2	thetao, sst, ts, ta, tauu	
15	IPSL-CM6A-LR	r1i1p1f1	thetao, sst, ts, ta, tauu, swr	thetao, sst, ts, ta, tauu, swr
16	MIROC-ES2L	r1i1p1f2	thetao, sst, ts, ta, tauu, swr	thetao, sst, ts, ta, tauu
17	MRI-ESM2-0	r1i1p1f1	thetao, sst, ts, ta, tauu, swr	thetao, sst, ts, ta, tauu, swr
